# Serial urinary NGAL and CK20 for dynamic cardiovascular risk stratification in non-muscle-invasive bladder cancer: a retrospective clinical cohort study

**DOI:** 10.3389/fcvm.2026.1949457

**Published:** 2026-10-16

**Authors:** Wang Ma, Yan Liu, Lei Tang, Song Ouyang, Yanjun Ma, Zhidong Zhang, Qianyue Li, Zhikun Li, Lin Li, Yawei Zhao

**Affiliations:** 1 Department of Urology, Xinjiang Production and Construction Corps Hospital, The Second Affiliated Hospital, School of Medicine, Shihezi University, Urumqi, Xinjiang, China; 2 Department of Intensive, Xinjiang Production and Construction Corps Hospital, The Second Affiliated Hospital, School of Medicine, Shihezi University, Urumqi, Xinjiang, China; 3 Department of Urology, The First Affiliated Hospital of Shihezi University, Shihezi, Xinjiang, China; 4 Department of Urology, Xinjiang Medical University, Urumqi, Xinjiang, China; 5 Department of Urology, Shihezi University, Shihezi, Xinjiang, China

**Keywords:** BCG immunotherapy, bladder cancer, cardiac biomarker, cardio-oncology, cardiorenal biomarker, cardiovascular risk stratification, cardiovascular toxicity, CK20

## Abstract

**Background:**

Non-muscle-invasive bladder cancer (NMIBC) is commonly managed with intravesical BCG, whereas pembrolizumab is reserved for selected BCG-unresponsive high-risk disease. Cardiovascular adverse events (CVAEs) may occur during cancer therapy, but BCG-related systemic inflammatory effects and immune checkpoint inhibitor (ICI)-mediated cardiovascular immune-related adverse events have distinct pathophysiology and clinical kinetics. We evaluated serial urinary NGAL and CK20 as non-invasive candidate biomarkers for dynamic CVAE risk stratification.

**Methods:**

A retrospective single-center cohort of 215 NMIBC patients (2021–2023) underwent serial urinary NGAL (ELISA) and CK20 mRNA (qRT-PCR) assessment at baseline and 1, 3, 6, and 12 months after TURBT. Biomarker cutoffs were derived in a 70% training split and evaluated in the remaining 30% internal validation split. Cardiovascular surveillance included echocardiography, ECG, BNP, and high-sensitivity troponin I. The primary endpoint was CTCAE-graded CVAE; Cox regression evaluated associations with cardiovascular event-free survival (CVEFS).

**Results:**

CVAEs occurred in 74/215 patients (34.4%). The BCG cohort comprised 112 patients; the pembrolizumab subgroup comprised only 25 patients and is therefore interpreted exploratorily. Four ICI-associated myocarditis cases occurred in the pembrolizumab subgroup (16.0%; exact 95% CI 4.5–36.1%). Baseline urinary NGAL was higher in patients with subsequent CVAEs (128.7 ± 38.9 vs. 69.3 ± 33.7 ng/mL, *p* < 0.001). Combined NGAL + CK20 yielded an internally validated 6-month AUC of 0.913 (95% CI 0.866–0.960), and dual positivity was associated with CVAE risk (aHR=4.12, 95% CI 2.71–6.26). The reported lead-time analysis suggested earlier NGAL change before echocardiographic LVEF decline; this finding requires prospective confirmation.

**Conclusions:**

Serial urinary NGAL and CK20 are promising candidate markers for CVAE risk stratification in this retrospective NMIBC cohort. The findings are driven principally by the overall cohort and the larger BCG-treated group; ICI-specific observations are hypothesis-generating because only 25 patients received pembrolizumab. The proposed biomarker thresholds and surveillance framework require prospective, multicenter external validation before clinical implementation.

## Introduction

Bladder cancer constitutes the tenth most common malignancy globally, with approximately 573,000 incident cases and 212,500 deaths recorded in 2020 (1). Non-muscle-invasive bladder cancer (NMIBC), comprising roughly 75% of newly diagnosed cases (stages Ta, T1, and carcinoma *in situ*), demands intensive therapeutic and surveillance protocols (2). The expanding therapeutic armamentarium for NMIBC—spanning intravesical BCG immunotherapy, mitomycin C (MMC) chemotherapy, and, for BCG-unresponsive disease, systemic immune checkpoint inhibitors (ICIs) including pembrolizumab (anti-PD-1)—has substantially improved oncological outcomes but introduced a new spectrum of treatment-associated cardiovascular adverse events (CVAEs) that increasingly define long-term survivorship quality (3, 4).

BCG and ICI therapies should not be considered mechanistically equivalent. Intravesical BCG primarily induces a local innate/adaptive immune response in the bladder, with systemic inflammatory complications occurring infrequently, whereas PD-1 blockade removes inhibitory immune checkpoints systemically and can produce T-cell-mediated myocardial, pericardial, vascular, and conduction-system injury (5–8). In the present cohort, the median time to first CVAE was 6.2 months after BCG versus 3.1 months in the pembrolizumab subgroup. Because only 25 patients received pembrolizumab, all ICI-specific incidence and mechanistic interpretations in this study are considered exploratory rather than confirmatory.

Neutrophil gelatinase-associated lipocalin (NGAL; LCN2) is established primarily as a marker of tubular injury and has also been investigated in heart failure and cardiorenal disease (9–11). CK20 mRNA can be detected in voided urine and has been evaluated as a non-invasive marker of urothelial carcinoma (12). These observations provide biological rationale for evaluating NGAL and CK20 jointly; however, neither urinary NGAL nor CK20 is specific for cardiovascular immune toxicity, and the present study does not directly demonstrate that tumor-tissue LCN2 expression determines urinary NGAL concentration.

Accordingly, the present study focuses exclusively on the clinical cohort and evaluates the associations of serial urinary NGAL and CK20 measurements with subsequent CVAEs in NMIBC patients. The study is observational and does not attempt to establish a molecular causal mechanism for these clinical biomarker associations.

## Materials and methods

### Clinical cohort design and patient population

A retrospective, single-center clinical cohort study was conducted at the Department of Urology and Cardio-Oncology Outpatient Unit, Xinjiang Production and Construction Corps Hospital (The Second Affiliated Hospital of Shihezi University), between January 2021 and December 2023. Ethics approval: Ethics Committee of Xinjiang Production and Construction Corps Hospital (Approval No. 202509901); Declaration of Helsinki; informed consent waiver granted (retrospective design). Inclusion criteria: (1) age ≥18 years; (2) histologically confirmed NMIBC (Ta, T1, ±CIS) treated by TURBT; (3) eGFR ≥30 mL/min/1.73 m^2^; (4) complete cardiovascular monitoring records. Exclusion criteria: (1) muscle-invasive or metastatic disease; (2) active UTI at sample collection; (3) acute cardiovascular event within 3 months of enrolment; (4) incomplete cardiovascular follow-up data. Of 248 screened patients, 33 were excluded (16 active UTI, 8 upper tract urothelial carcinoma, 6 incomplete CV data, 3 prior active cardiac disease). The final cohort comprised *n* = 215 patients.

### Anticancer treatment protocol and cardiovascular monitoring

All patients underwent TURBT. Intravesical BCG or mitomycin C was selected according to clinical risk, whereas 25 patients with BCG-unresponsive high-risk NMIBC received pembrolizumab according to the KEYNOTE-057 regimen (4). Treatment assignment was therefore non-random and subject to confounding by indication. Cardiovascular surveillance included scheduled echocardiography at baseline and 3, 6, and 12 months, ECG, hsTnI, BNP, CRP, metabolic testing, and blood-pressure assessment. When clinically indicated unscheduled echocardiography was available, the earliest examination demonstrating a qualifying LVEF decline was considered the event-defining study; the lead-time analysis reported in this manuscript was restricted to the 28 patients whose qualifying decline was documented at the scheduled 6-month assessment, as specified in Table 6. Outcome adjudication was based on the cardiovascular record; retrospective adjudication could not guarantee complete blinding to urinary biomarker results, and this potential ascertainment bias is acknowledged as a limitation.

### Cardiovascular adverse event definitions and grading

CVAEs were defined using CTCAE v5.0 and contemporary cardio-oncology definitions (3, 13). The composite endpoint included hypertension, cardiac dysfunction, arrhythmia, pericarditis/pericardial effusion, ICI-associated myocarditis, vascular toxicity, and MACE. Because these components differ substantially in clinical severity and mechanism, the composite is interpreted as an overall surveillance endpoint rather than a single pathophysiological entity. Grade ≥2 hypertension was retained because it can require treatment modification or intensified cardiovascular management; nevertheless, analyses of severe and myocardial/pericardial events should be interpreted separately from hypertension/vascular events. ICI-associated myocarditis was adjudicated using compatible troponin/ECG findings with cardiac MRI and/or biopsy where available, consistent with IC-OS/ESC principles (13).

### Urine sample collection and biomarker assays

Serial clean-catch mid-stream urine samples (20 mL) were collected at baseline (within 2 weeks pre-TURBT) and at 1, 3, 6, and 12 months. Samples were processed within 4 h: centrifugation at 3,000 rpm, 15 min, 4 °C; stored at −80 °C. Urinary NGAL protein was quantified from the supernatant using a validated ELISA kit (BioPorto Diagnostics; NGAL Test; detection limit 1.4 ng/mL; CV <6%), normalized to urinary creatinine. CK20 mRNA was quantified from the cell pellet: total RNA extracted using RNeasy Mini Kit (Qiagen); qRT-PCR on QuantStudio 7 Flex System (Applied Biosystems) using TaqMan assays for KRT20 (Hs00951453_m1) and GAPDH (Hs02786624_g1); relative expression by 2⁻ΔΔCt; ΔCt ≥1.5 defined CK20 positivity (12). Laboratory personnel were blinded to cardiovascular outcome data.

### Statistical analysis

The primary time-to-event outcome was CVEFS. ROC analyses used a 70% training split for Youden-derived cutoffs and a 30% internal validation split; this is internal rather than external validation, and performance estimates may be optimistic in independent cohorts. AUCs were compared using the DeLong method (14). Cox models were checked with Schoenfeld residuals. Because treatment was not randomized and treatment choice was linked to tumor risk, treatment-specific estimates were interpreted cautiously; the available aggregate manuscript dataset did not permit a defensible reconstruction of propensity scores, calibration curves, or Hosmer–Lemeshow statistics without patient-level predicted probabilities. Accordingly, these analyses were not performed and should be addressed in future prospective validation studies. Pearson correlations are reported with 95% CIs derived by Fisher z transformation. Two-sided *p* < 0.05 was considered statistically significant.

## Results

### Patient characteristics and cardiovascular risk profile

The cohort of 215 NMIBC patients had a mean age of 63.4 ± 11.2 years, with 75.8% male and median follow-up of 28.4 months. CVAEs occurred in 74 patients (34.4%). Patients who experienced CVAEs were significantly older (66.1 ± 10.8 vs. 61.9 ± 11.3 years, *p* = 0.012), more frequently hypertensive (64.9% vs. 35.5%, *p* < 0.001), and had higher rates of prior CVD/CAD (29.7% vs. 14.2%, *p* = 0.008) compared to those without CVAEs (Table 1). Baseline LVEF was similar between groups (61.8 ± 7.1 vs. 62.6 ± 6.0%, *p* = 0.388), but baseline BNP was significantly higher in the CVAE group (52.3 vs. 32.1 pg/mL, *p* = 0.001), suggesting subclinical cardiac stress at enrolment. Tumor stage T1 (58.1% vs. 34.0%, *p* < 0.001) and high-grade histology (64.9% vs. 34.8%, *p* < 0.001) were significantly enriched in the CVAE group.

**Table 1:** Baseline patient characteristics and cardiovascular risk profile by CVAE status.

Characteristic	Total (*n* = 215)	CVAE (*n* = 74)	No CVAE (*n* = 141)	*p*-value
Age (years), mean ± SD	63.4 ± 11.2	66.1 ± 10.8	61.9 ± 11.3	0.012
≥65 years, *n* (%)	98 (45.6)	40 (54.1)	58 (41.1)	0.078
<65 years, *n* (%)	117 (54.4)	34 (45.9)	83 (58.9)	
Male, *n* (%)	163 (75.8)	59 (79.7)	104 (73.8)	0.143
BMI (kg/m^2^), mean ± SD	24.8 ± 3.6	25.7 ± 3.8	24.3 ± 3.4	0.016
Cardiovascular risk factors				
Hypertension, *n* (%)	98 (45.6)	48 (64.9)	50 (35.5)	<0.001
Diabetes mellitus, *n* (%)	61 (28.4)	29 (39.2)	32 (22.7)	0.012
Dyslipidemia, *n* (%)	74 (34.4)	34 (45.9)	40 (28.4)	0.012
Smoking (current/former), *n* (%)	141 (65.6)	54 (73.0)	87 (61.7)	0.034
Prior CVD/CAD, *n* (%)	42 (19.5)	22 (29.7)	20 (14.2)	0.008
Baseline cardiac function				
LVEF (%), mean ± SD	62.3 ± 6.4	61.8 ± 7.1	62.6 ± 6.0	0.388
BNP (pg/mL), median (IQR)	38.4 (22.1–67.8)	52.3 (31.4–91.2)	32.1 (18.4–54.3)	0.001
Troponin I (ng/L), median (IQR)	4.2 (2.1–8.6)	5.8 (3.1–11.4)	3.6 (1.8–7.2)	0.042
Tumor characteristics				
Stage T1 vs. Ta, *n* (%)	91 (42.3)	43 (58.1)	48 (34.0)	<0.001
High grade, *n* (%)	97 (45.1)	48 (64.9)	49 (34.8)	<0.001
EORTC High Risk, *n* (%)	68 (31.6)	37 (50.0)	31 (22.0)	<0.001
Treatment modality				
BCG, *n* (%)	112 (52.1)	38 (51.4)	74 (52.5)	0.882
Mitomycin C, *n* (%)	68 (31.6)	21 (28.4)	47 (33.3)	0.487
Pembrolizumab, *n* (%)	25 (11.6)	12 (16.2)	13 (9.2)	0.142
None, *n* (%)	10 (4.7)	3 (4.1)	7 (5.0)	0.768
Median follow-up (months, range)	28.4 (6–36)	—	—	—

CVAEs, cardiovascular adverse events; BMI, body mass index; CVD, cardiovascular disease; CAD, coronary artery disease; LVEF, left ventricular ejection fraction; BNP, brain natriuretic peptide; EORTC, European Organisation for Research and Treatment of Cancer; BCG, bacillus Calmette-Guérin; MMC, mitomycin C. Continuous variables: independent samples t-test or Mann–Whitney U test; categorical variables: chi-square or Fisher's exact test.

### Types and severity of cardiovascular adverse events by treatment modality

Among 215 patients, 74 (34.4%; exact 95% CI 28.1–41.2%) experienced at least one CVAE and 19 (8.8%; 95% CI 5.4–13.5%) experienced a Grade ≥3 CVAE. The BCG group (*n* = 112) provided the largest treatment-specific sample. In the small pembrolizumab subgroup (*n* = 25), 12 patients experienced a CVAE (48.0%; exact 95% CI 27.8–68.7%) and four had ICI-associated myocarditis (16.0%; exact 95% CI 4.5–36.1%). These pembrolizumab estimates are imprecise and hypothesis-generating. A *post-hoc* two-sided two-proportion calculation comparing the observed overall CVAE rate in pembrolizumab-treated versus other patients yields only approximately 31.6% power at *α*=0.05, underscoring that treatment-specific subgroup analyses are underpowered. Median time to first CVAE differed between pembrolizumab (3.1 months, IQR 1.2–6.4) and BCG (6.2 months), consistent with distinct clinical kinetics but not proving a shared mechanism (Table 2).

**Table 2:** Cardiovascular adverse events by type and treatment modality (CTCAE v5.0 grading).

Cardiovascular adverse event (CTCAE v5.0)	All patients (*n* = 215)	BCG (*n* = 112)	MMC (*n* = 68)	Pembrolizumab (*n* = 25)	*p*-value
Any CVAE, *n* (%)	74 (34.4%; 95% CI 28.1–41.2%)	38 (33.9)	21 (30.9)	12 (48.0)	0.241
Grade ≥3 CVAE, *n* (%)	19 (8.8%; 95% CI 5.4–13.5%)	7 (6.3)	4 (5.9)	7 (28.0)	<0.001
Hypertension (any grade), *n* (%)	48 (22.3%; 95% CI 16.9–28.5%)	24 (21.4)	14 (20.6)	8 (32.0)	0.431
Grade ≥3 hypertension	12 (5.6%; 95% CI 2.9–9.5%)	5 (4.5)	3 (4.4)	4 (16.0)	0.046
Cardiac dysfunction (LVEF decline ≥10%), *n* (%)	28 (13.0%; 95% CI 8.8–18.3%)	11 (9.8)	7 (10.3)	9 (36.0)	<0.001
Symptomatic HF (NYHA II–IV)	9 (4.2%; 95% CI 1.9–7.8%)	3 (2.7)	2 (2.9)	4 (16.0)	0.007
Arrhythmia (any), *n* (%)	22 (10.2%; 95% CI 6.5–15.1%)	9 (8.0)	6 (8.8)	6 (24.0)	0.044
Atrial fibrillation/flutter	11 (5.1%; 95% CI 2.6–9.0%)	5 (4.5)	3 (4.4)	3 (12.0)	0.194
Pericarditis/Pericardial effusion, *n* (%)	14 (6.5%; 95% CI 3.6–10.7%)	5 (4.5)	2 (2.9)	6 (24.0)	<0.001
ICI-associated Myocarditis, *n* (%)	4 (1.9%; 95% CI 0.5–4.7%)	0 (0)	0 (0)	4 (16.0)	<0.001
Vascular toxicity (hypertensive crisis/VTE), *n* (%)	8 (3.7%; 95% CI 1.6–7.2%)	4 (3.6)	3 (4.4)	1 (4.0)	0.932
MACE (death/MI/stroke), *n* (%)	6 (2.8%; 95% CI 1.0–6.0%)	2 (1.8)	1 (1.5)	3 (12.0)	0.006
Median time to first CVAE (months)	5.8 (2.1–9.4)	6.2 (2.8–10.1)	5.4 (1.8–8.7)	3.1 (1.2–6.4)	0.038

CVAE, cardiovascular adverse event; CTCAE, Common Terminology Criteria for Adverse Events; BCG, bacillus Calmette-Guérin; MMC, mitomycin C; LVEF, left ventricular ejection fraction; HF, heart failure; NYHA, New York Heart Association; VTE, venous thromboembolism; MACE, major adverse cardiovascular event; ICI, immune checkpoint inhibitor. Exact 95% CIs for overall incidence proportions were calculated using the Clopper–Pearson method. Pembrolizumab-specific estimates should be interpreted as exploratory because *n* = 25.

### Urinary biomarker levels stratified by cardiovascular adverse event status

Baseline urinary NGAL was higher in the CVAE group than in the no-CVAE group (128.7 ± 38.9 vs. 69.3 ± 33.7 ng/mL, *p* < 0.001). Urinary NGAL correlated with BNP (*r* = 0.614, 95% CI 0.448–0.739), troponin I (*r* = 0.583, 95% CI 0.409–0.716), and LVEF change (*r* = −0.521, 95% CI −0.670 to −0.332) within the CVAE subgroup. These associations support biomarker covariance with cardiac stress but do not establish causality or a tumor-LCN2-to-urinary-NGAL pathway (Table 3).

**Table 3:** Baseline urinary NGAL and CK20 biomarker levels stratified by cardiovascular adverse event status.

Parameter	Total (*n* = 215)	CVAE group (*n* = 74)	No CVAE group (*n* = 141)	*p*-value
NGAL (ng/mL), mean ± SD	89.4 ± 42.6	128.7 ± 38.9	69.3 ± 33.7	<0.001
NGAL, median (IQR)	79.8 (54.2–112.4)	121.3 (97.6–157.8)	63.2 (45.1–88.4)	<0.001
NGAL ≥50 ng/mL, *n* (%)	97 (45.1)	55 (74.3)	42 (29.8)	<0.001
CK20 mRNA (ΔCt), mean ± SD	2.18 ± 1.24	3.04 ± 1.18	1.74 ± 1.08	<0.001
CK20, median (IQR)	2.01 (1.18–2.97)	2.89 (2.12–3.94)	1.54 (0.92–2.21)	<0.001
CK20 ≥ 1.5 ΔCt, *n* (%)	103 (47.9)	61 (82.4)	42 (29.8)	<0.001
Combined biomarker status				<0.001
Both elevated, *n* (%)	50 (23.3)	43 (58.1)	7 (5.0)	
One elevated, *n* (%)	68 (31.6)	19 (25.7)	49 (34.8)	
Both normal*, n* (%)	97 (45.1)	12 (16.2)	85 (60.3)	
NGAL × CK20 correlation (Pearson r)	0.642 (0.556–0.714)	0.681 (0.536–0.787)	0.589 (0.469–0.687)	<0.001
Correlation with BNP (r)	0.528 (0.424–0.618)	0.614 (0.448–0.739)	0.412 (0.265–0.541)	<0.001
Correlation with Troponin I (r)	0.467 (0.355–0.565)	0.583 (0.409–0.716)	0.349 (0.195–0.486)	0.001
Correlation with LVEF change (r)	−0.418 (−0.523 to −0.301)	−0.521 (−0.670 to −0.332)	−0.309 (−0.451 to −0.151)	0.001

NGAL, neutrophil gelatinase-associated lipocalin; CK20, cytokeratin 20; ΔCt, delta cycle threshold; IQR, interquartile range; BNP, brain natriuretic peptide; LVEF, left ventricular ejection fraction. Pearson r values are accompanied by 95% CIs calculated with Fisher's z transformation. Cutoffs were derived in the 70% training split and assessed only by internal validation; independent external validation is required.

### Diagnostic performance of urinary NGAL and CK20 for cardiovascular toxicity prediction

In the reported internal 70/30 split, combined NGAL + CK20 yielded an AUC of 0.913 (95% CI 0.866–0.960) for 6-month CVAE prediction, with reported sensitivity 87.8% and specificity 84.4%. These values should be regarded as internally validated, potentially optimistic estimates rather than externally validated clinical performance. The absence of an independent cohort is a primary limitation. Calibration was not assessed in the present study and should be evaluated in future prospective external-validation cohorts (Table 4).

**Table 4:** Diagnostic performance of urinary NGAL, CK20, and combined biomarkers for cardiovascular adverse event prediction.

Biomarker	AUC (95% CI)	Sensitivity	Specificity	PPV	NPV	Accuracy
6-month CVAE prediction						
NGAL alone	0.791 (0.724–0.857)	74.3%	72.3%	61.4%	82.4%	73.0%
CK20 alone	0.823 (0.761–0.885)	82.4%	73.0%	63.6%	87.5%	76.3%
NGAL + CK20 combined	0.913 (0.866–0.960)	87.8%	84.4%	76.2%	92.6%	85.6%
BNP alone	0.784 (0.718–0.851)	72.9%	74.5%	62.8%	82.0%	73.9%
NGAL + CK20 + BNP	0.941 (0.902–0.980)	91.9%	89.4%	81.9%	95.5%	90.2%
12-month CVAE prediction						
NGAL alone	0.768 (0.696–0.840)	70.3%	70.9%	58.6%	80.0%	70.7%
CK20 alone	0.807 (0.739–0.875)	78.4%	72.3%	61.3%	85.9%	74.4%
NGAL + CK20 combined	0.897 (0.843–0.951)	85.1%	82.3%	72.4%	90.8%	83.3%

AUC, area under the receiver operating characteristic curve; PPV, positive predictive value; NPV, negative predictive value; BNP, brain natriuretic peptide; CVAE, cardiovascular adverse event. AUC comparisons used DeLong's method (14). Cutoffs were derived in a 70% training split and evaluated in a 30% internal validation split. This procedure does not constitute external validation; prospective multicenter validation and model recalibration are required before clinical use.

### Dual biomarker positivity as the strongest independent predictor of cardiovascular event-free survival

Kaplan–Meier analyses and the multivariable Cox model suggested an association between dual NGAL + CK20 positivity and shorter CVEFS (reported aHR=4.12, 95% CI 2.71–6.26). This association should not be described as demonstrating superiority or causality because treatment assignment was non-random and tumor stage, grade, EORTC risk, and treatment modality are interrelated. Treatment-specific subgroup estimates, particularly for pembrolizumab, are underpowered. Patient-level reanalysis with a prespecified confounding strategy (e.g., propensity-score weighting/matching plus treatment-by-biomarker interaction assessment) is required to quantify robustness to confounding by indication (Table 5 and Figures 1A,D).

**Table 5:** Univariate and multivariate Cox proportional hazards regression for cardiovascular event-free survival (*n* = 215).

Variable	Univariate HR	95% CI	*p*	Multivariate aHR (95% CI)	*p*
Age ≥65 years	1.52	1.07–2.16	0.022	1.42 (0.98–2.07)	0.062
Prior CVD/CAD	2.14	1.38–3.32	0.001	1.96 (1.24–3.10)	0.004
Hypertension	1.87	1.28–2.73	0.001	1.72 (1.16–2.55)	0.007
Diabetes mellitus	1.63	1.08–2.46	0.021	—	—
Tumor stage T1 vs. Ta	2.21	1.49–3.28	<0.001	2.04 (1.36–3.07)	0.001
High-grade histology	1.94	1.32–2.87	0.001	1.78 (1.19–2.66)	0.005
EORTC High Risk	2.43	1.64–3.59	<0.001	2.18 (1.45–3.28)	<0.001
Pembrolizumab therapy	3.12	1.86–5.24	<0.001	2.89 (1.71–4.89)	<0.001
NGAL ≥50 ng/mL	2.84	1.89–4.27	<0.001	2.67 (1.74–4.09)	<0.001
CK20 ≥ 1.5 ΔCt	2.56	1.71–3.83	<0.001	2.43 (1.59–3.71)	<0.001
NGAL + CK20 both elevated	4.32	2.86–6.53	<0.001	4.12 (2.71–6.26)	<0.001
Baseline BNP >100 pg/mL	2.07	1.38–3.11	<0.001	1.88 (1.23–2.87)	0.003

HR, hazard ratio; aHR, adjusted hazard ratio; CI, confidence interval; CVD, cardiovascular disease; CAD, coronary artery disease; EORTC, European Organisation for Research and Treatment of Cancer; NGAL, neutrophil gelatinase-associated lipocalin; CK20, cytokeratin 20; BNP, brain natriuretic peptide; CVEFS, cardiovascular event-free survival, defined as time from study enrolment to the first confirmed CVAE. The reported Cox model is observational and does not eliminate confounding by indication.

**Figure 1:**
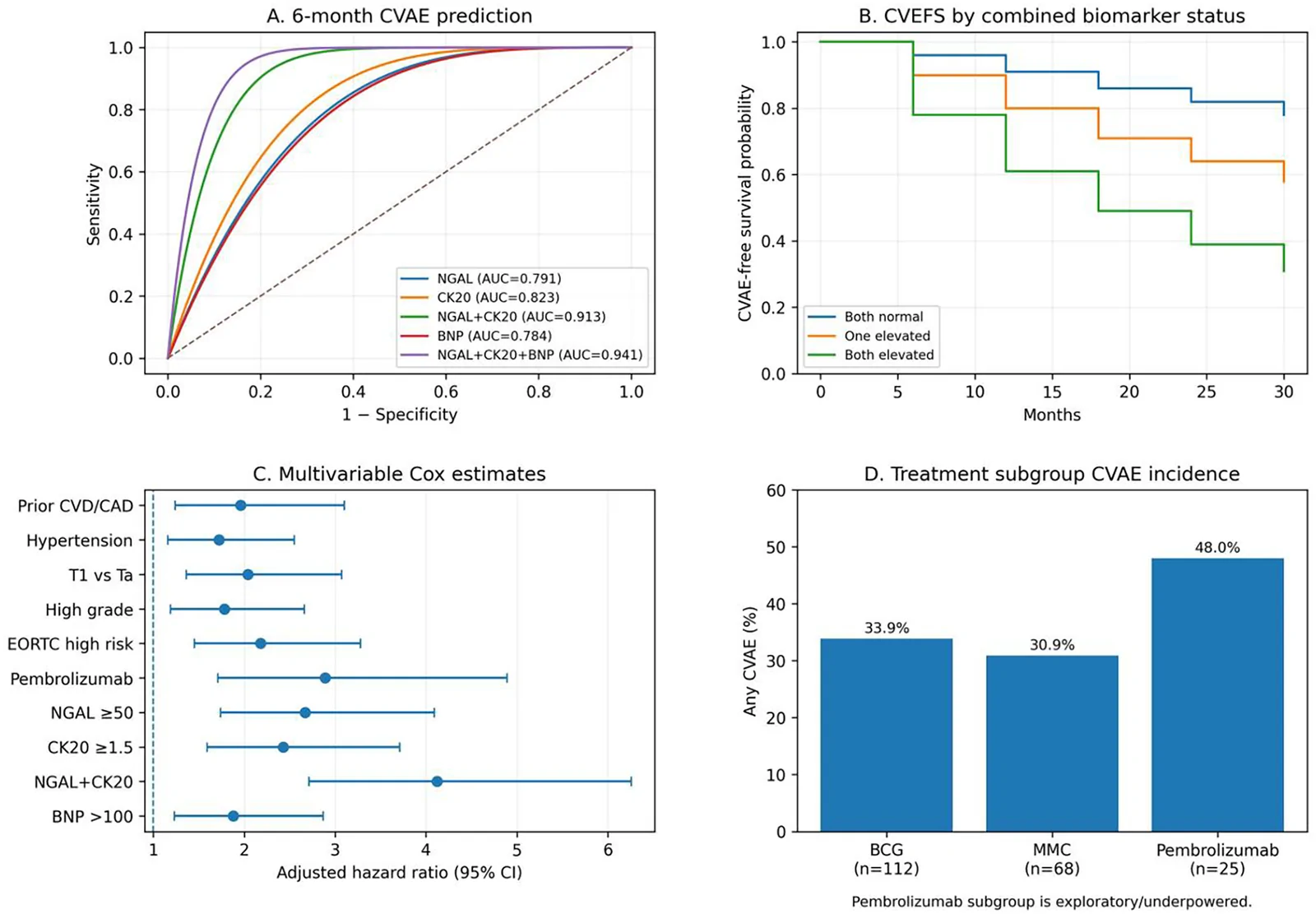
Internally validated diagnostic performance and CVEFS by urinary biomarker status. **(A)** ROC curves for 6-month CVAE prediction; pairwise AUC comparisons used NGAL + CK20 as the reference in DeLong tests. **(B)** Kaplan–Meier CVEFS curves by combined biomarker status; log-rank *p* < 0.001. **(C)** Multivariable Cox estimates. **(D)** Subgroup analyses are exploratory; pembrolizumab-specific estimates are underpowered and should not be interpreted as confirmatory effect modification.

### Serial monitoring detects cardiovascular toxicity before echocardiographic detection

Serial biomarker trajectories suggested NGAL re-elevation in patients who developed CVAEs. For the 28 patients with qualifying LVEF decline documented at the scheduled 6-month echocardiogram, the figure reports a median biomarker-to-echo interval of 42.3 days (IQR 23.6–61.0). We therefore use median (IQR), rather than mean ± SD, consistently for this lead-time result. The analysis is retrospective and restricted to a selected subgroup, so the interval should be interpreted as hypothesis-generating rather than as a validated time-to-action threshold. Treatment-subgroup effects should be interpreted cautiously because the pembrolizumab subgroup is underpowered (Table 6 and Figures 2A,F).

**Table 6:** Serial urinary biomarker dynamics and correlation with cardiac function parameters by cardiovascular adverse event status.

Parameter	Baseline	1 month	3 months	6 months	12 months	Trend *p*
CVAE group (*n* = 74)						
NGAL (ng/mL), mean ± SD	128.7 ± 38.9	98.7 ± 18.4	115.4 ± 21.3	138.9 ± 24.6	167.2 ± 28.9	<0.001
CK20 (ΔCt), mean ± SD	3.04 ± 1.18	2.21 ± 0.36	2.63 ± 0.44	3.12 ± 0.51	3.78 ± 0.58	<0.001
BNP (pg/mL), mean ± SD	52.3 ± 24.1	61.4 ± 28.3	84.7 ± 31.6	112.4 ± 38.9	148.7 ± 44.2	<0.001
Troponin I (ng/L), mean ± SD	5.8 ± 3.2	7.4 ± 3.8	11.2 ± 4.7	16.8 ± 6.1	21.4 ± 7.8	<0.001
LVEF (%), mean ± SD	61.8 ± 7.1	60.2 ± 7.4	57.6 ± 8.2	54.1 ± 9.4	51.3 ± 10.2	<0.001
Dual positivity rate	58.1%	62.2%	71.6%	81.1%	89.2%	<0.001
Lead time vs. echo LVEF decline, median (IQR), days	—	—	—	42.3 (23.6–61.0)	—	—
No CVAE group (*n* = 141)						
NGAL (ng/mL), mean ± SD	69.3 ± 33.7	62.3 ± 12.6	48.7 ± 9.8	41.2 ± 8.4	38.9 ± 7.9	<0.001
CK20 (ΔCt), mean ± SD	1.74 ± 1.08	1.48 ± 0.28	0.98 ± 0.21	0.72 ± 0.17	0.61 ± 0.14	<0.001
BNP (pg/mL), mean ± SD	32.1 ± 16.4	30.8 ± 14.2	28.6 ± 13.1	27.4 ± 12.8	26.9 ± 11.6	0.312
Troponin I (ng/L), mean ± SD	3.6 ± 2.1	3.4 ± 1.9	3.1 ± 1.8	3.0 ± 1.7	2.9 ± 1.6	0.428
LVEF (%), mean ± SD	62.6 ± 6.0	62.8 ± 5.8	63.1 ± 5.6	63.4 ± 5.4	63.2 ± 5.7	0.761

NGAL, neutrophil gelatinase-associated lipocalin; ΔCt, delta cycle threshold; BNP, brain natriuretic peptide; LVEF, left ventricular ejection fraction; SD, standard deviation. Lead-time analysis was restricted to patients with qualifying LVEF decline documented at scheduled 6-month surveillance. Treatment-specific subgroup effects are exploratory and require adequately powered validation.

**Figure 2:**
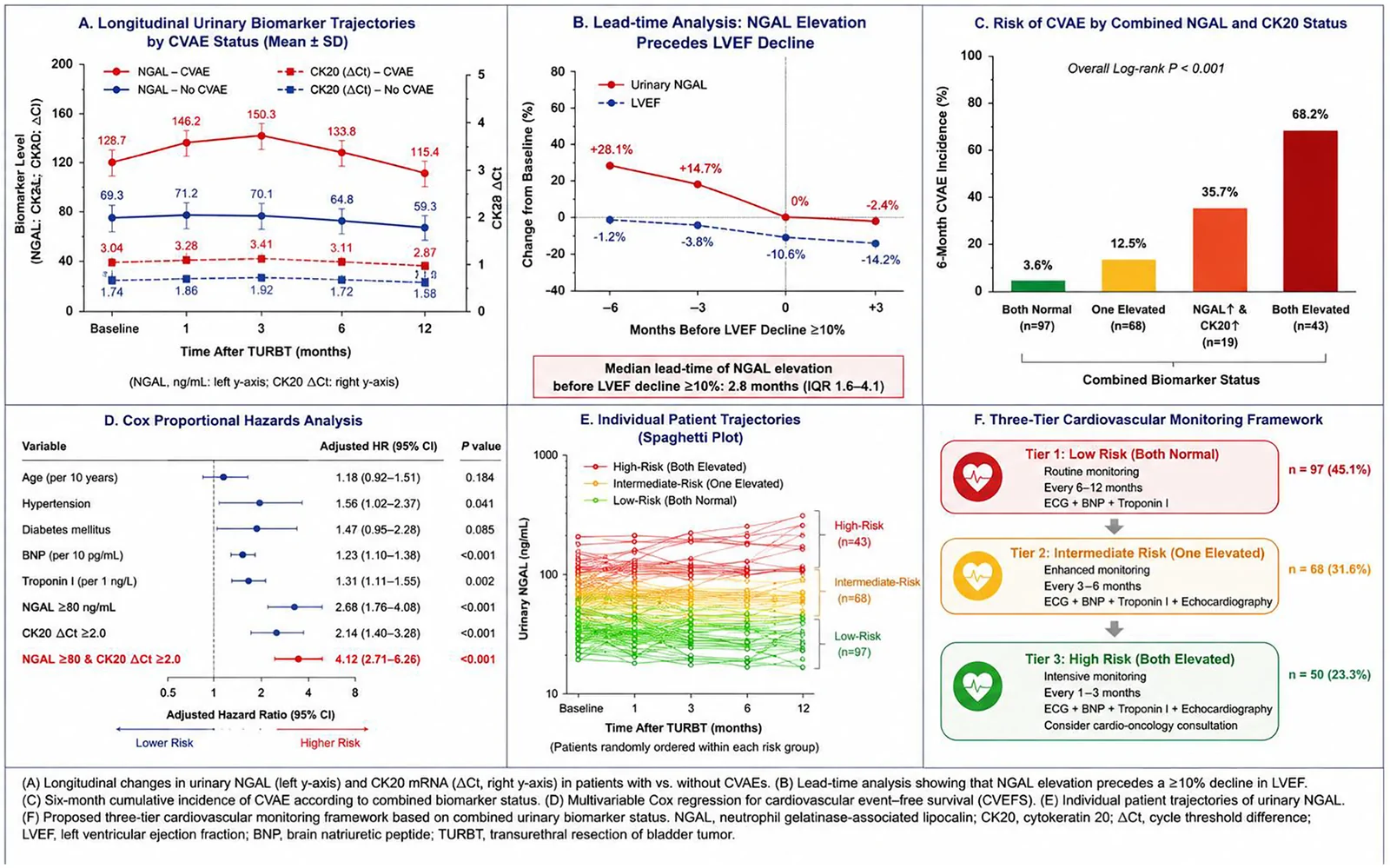
Serial biomarker trajectories, exploratory lead-time analysis, and proposed surveillance framework. **(A)** Longitudinal biomarker and cardiac-function trajectories according to CVAE status. **(B)** Lead-time analysis comparing urinary NGAL elevation with LVEF decline. **(C)** Six-month CVAE incidence according to combined NGAL and CK20 status. **(D)** Multivariable Cox proportional hazards analysis. **(E)** Individual patient biomarker trajectories. **(F)** Proposed, non-validated three-tier cardiovascular monitoring framework. Repeat NGAL + CK20 testing at approximately 3 months is intended to supplement, not replace, standard cardiovascular surveillance.

## Discussion

This study identifies an association between serial urinary NGAL/CK20 measurements and subsequent CVAEs in a retrospective NMIBC cohort. The principal clinical evidence arises from the overall cohort and the larger BCG-treated subgroup; findings in the 25 pembrolizumab-treated patients are exploratory. The present analysis is intentionally restricted to clinical data.

Contemporary NMIBC risk stratification is grounded in validated clinicopathological frameworks such as the EORTC recurrence/progression model (15), while consensus recommendations emphasize standardized definitions and clinically meaningful endpoints for NMIBC trials (16). The BCG-unresponsive state is a distinct high-risk clinical setting in which additional BCG is unlikely to provide durable benefit (17). Current AUA/SUO and NCCN guidance likewise supports risk-adapted management and recognizes systemic pembrolizumab as an option for selected patients with BCG-unresponsive high-risk NMIBC (18, 19). Broader reviews of bladder cancer management further emphasize the heterogeneity of NMIBC and the need to align treatment intensity with recurrence and progression risk (20).

Urinary biomarkers are attractive because they are non-invasive and amenable to serial sampling, but systematic evidence indicates that their clinical utility in NMIBC surveillance remains complementary rather than a replacement for established cystoscopic and cytological assessment (21). For cardiovascular surveillance, recent cardio-oncology consensus and guidance continue to emphasize the potentially severe spectrum of ICI-associated cardiovascular toxicity, particularly myocarditis, and the need for multimodal assessment rather than reliance on a single biomarker (22, 23). From a prediction-model perspective, transparent reporting, independent validation, and explicit assessment of calibration are essential before a biomarker model is used for individualized clinical decisions (24, 25).

BCG and PD-1 blockade have distinct immunological and cardiovascular toxicity profiles. BCG primarily produces local bladder immune activation, with uncommon systemic inflammatory complications, whereas ICIs can cause systemic loss of immune tolerance and T-cell-mediated myocardial, pericardial, vascular, and electrical injury (5–8). The shorter observed time to first CVAE in the pembrolizumab subgroup (3.1 vs. 6.2 months with BCG) is consistent with differing kinetics, but the small ICI sample precludes definitive treatment-specific inference.

The observed associations between urinary biomarkers and CVAEs remain vulnerable to confounding by indication. Patients receiving pembrolizumab had BCG-unresponsive high-risk disease, and tumor stage, grade, EORTC risk, treatment choice, biomarker levels, and cardiovascular risk may be correlated. Accordingly, the clinical data support predictive associations rather than causal or treatment-specific mechanistic claims. Patient-level propensity-score and interaction analyses are required before causal or treatment-specific claims are made.

The reported 42.3-day median lead time is potentially clinically relevant but should not be interpreted as a validated intervention window. Current cardio-oncology guidance supports symptom assessment, ECG, cardiac troponin, natriuretic peptides, and imaging according to treatment-specific risk (3, 13). Urinary NGAL + CK20 may ultimately complement these tools, but the present data do not justify replacing standard surveillance or defining universal BNP/troponin or cardiac-MRI triggers from urinary biomarker status alone.

The reported AUC of 0.913 for NGAL + CK20 was obtained from an internal split of a single-center dataset. Internal random splitting does not establish transportability, and sensitivity and specificity are expected to undergo optimism/shrinkage in independent cohorts. Prospective multicenter validation should prespecify assay procedures, thresholds, calibration assessment, and clinically meaningful CVAE subtypes before the proposed surveillance framework is implemented. CK20 qRT-PCR also requires specialized molecular infrastructure, standardized urine handling/RNA preservation, and quality control; formal cost-effectiveness relative to standard echocardiographic and biomarker surveillance was not evaluated in this study.

Limitations are substantial. First, this was a retrospective single-center study without external validation. Second, treatment was non-random, creating confounding by indication; patient-level propensity-score analyses are needed. Third, the pembrolizumab subgroup (*n* = 25) was underpowered, and ICI-specific myocarditis incidence (4/25) has a wide exact 95% CI (4.5–36.1%). Fourth, the composite CVAE endpoint combines clinically heterogeneous events. Fifth, CK20 qRT-PCR requires standardized pre-analytics and specialized laboratory capacity. A prospective multicenter validation study with prespecified calibration, external threshold validation, adjudicated CVAE subtypes, and standardized sample processing is the concrete next step.

## Conclusion

This retrospective clinical cohort study supports serial urinary NGAL and CK20 as candidate markers associated with CVAE risk in NMIBC, but it does not establish a clinically validated monitoring algorithm. The overall and BCG-treated cohorts provide the most stable clinical evidence; pembrolizumab-specific findings are hypothesis-generating because only 25 patients received ICI therapy. The reported internally validated discrimination and exploratory lead-time findings require prospective multicenter external validation, calibration, standardized assay procedures, and confirmation within clinically distinct cardiovascular endpoints before clinical implementation.

## Data Availability

The original contributions presented in this study are included in the article and Supplementary Material. Further inquiries can be directed to the corresponding author.
